# Methods of Inactivation of SARS-CoV-2 for Downstream Biological Assays

**DOI:** 10.1093/infdis/jiaa507

**Published:** 2020-08-15

**Authors:** Edward I Patterson, Tessa Prince, Enyia R Anderson, Aitor Casas-Sanchez, Shirley L Smith, Cintia Cansado-Utrilla, Tom Solomon, Michael J Griffiths, Álvaro Acosta-Serrano, Lance Turtle, Grant L Hughes

**Affiliations:** 1 Department of Vector Biology and Tropical Disease Biology, Centre for Neglected Tropical Disease, Liverpool School of Tropical Medicine, Liverpool, United Kingdom; 2 National Institute for Health Research Health Protection Unit in Emerging and Zoonotic Infections, Department of Clinical Infection, Microbiology and Immunology, University of Liverpool, Liverpool, United Kingdom; 3 Institute of Infection and Global Health, University of Liverpool, Liverpool, United Kingdom; 4 Walton Centre NHS Foundation Trust, Liverpool, United Kingdom; 5 Department of Clinical Infection, Microbiology and Immunology, Institute of Infection and Global Health, University of Liverpool, Liverpool, United Kingdom; 6 Department of Neurology, Alder Hey Children’s NHS Trust, Liverpool, United Kingdom; 7 Tropical and Infectious Disease Unit, Liverpool University Hospitals Foundation NHS Trust, Liverpool, United Kingdom

**Keywords:** SARS-Cov-2, inactivation, temperature, detergents, methanol, paraformaldehyde, Trizol

## Abstract

The scientific community has responded to the coronavirus disease 2019 (COVID-19) pandemic by rapidly undertaking research to find effective strategies to reduce the burden of this disease. Encouragingly, researchers from a diverse array of fields are collectively working towards this goal. Research with infectious severe acute respiratory syndrome coronavirus 2 (SARS-CoV-2) is undertaken in high-containment laboratories; however, it is often desirable to work with samples at lower-containment levels. To facilitate the transfer of infectious samples from high-containment laboratories, we have tested methods commonly used to inactivate virus and prepare the sample for additional experiments. Incubation at 80°C, a range of detergents, Trizol reagents, and UV energies were successful at inactivating a high titer of SARS-CoV-2. Methanol and paraformaldehyde incubation of infected cells also inactivated the virus. These protocols can provide a framework for in-house inactivation of SARS-CoV-2 in other laboratories, ensuring the safe use of samples in lower-containment levels.

## Teaser

SARS-CoV-2 research requires the safe use of samples in low-containment laboratories. Heat, UV, and chemical reagents inactivated high titers of virus in solution and cells. These methods will facilitate use of once-infectious samples in downstream procedures.

The novel coronavirus, severe acute respiratory syndrome coronavirus 2 (SARS-CoV-2) emerged in December 2019 in Wuhan, China, and spread to the rest of the world in a few months causing a pandemic [1, 2]. This virus causes the coronavirus disease, known as COVID-19, in humans and, as of 17 May 2020, has infected almost 5 000 000 people and caused over 300 000 deaths [3]. Research on SARS-CoV-2 has increased exponentially since the beginning of the pandemic and will likely continue growing until an effective vaccine is developed. In the UK, and many other countries, SARS-CoV-2 is classified as a hazard group 3 pathogen. For handling clinical samples and performing experiments involving SARS-CoV-2 and other viruses in general, inactivation methods are needed in order to work under safe conditions. Additionally, the inactivation of the virus allows the transfer of the material from a containment level (CL) 3 to a CL2 laboratory, facilitating the performance of experiments and increasing the number of laboratories and researchers that can perform those experiments. Several methods of inactivation are available, but because this is a novel virus, the effectiveness of many of these methods on SARS-CoV-2 has not been tested yet. Some inactivation approaches have been tested on SARS-CoV, a coronavirus which spread between November 2002 and September 2003 and whose genome presents an 80% shared identity with the new SARS-CoV-2 [4]. It is expected that the outcome of both physical and chemical inactivation methods used against SARS-CoV-2 will be similar to SARS-CoV, but methods need to be validated prior to use of the new virus isolate.

Several methods for virus inactivation are available and the choice of which approach to use is often related to their compatibility with downstream applications. Heat inactivation has been used for several viruses [5, 6] and is a common method employed for antigen preservation of viral and bacterial pathogens. To preserve proteins in the sample that are related to host immune response, detergents and UV can be used to inactivate viruses [7, 8]. Detergents and Trizol are common additives in reagents used for virus inactivation, as well as RNA extraction from a range of sample types. Fixation of infected cells using methanol or paraformaldehyde is commonly used to preserve and stabilize cell morphology and can simultaneously render samples noninfectious [9]. UV irradiation, which inactivates viruses by modifying their nucleic acid structure, has been used successfully to inactivate many viruses, and in particular SARS-CoV [10]. Inactivation of SARS-CoV-2 through the use of UV would allow the safe use of the virus within a CL2 laboratory and prevent the possibility of laboratory-acquired infections. Here we aim to assess and describe physical and chemical inactivation protocols of SARS-CoV-2.

## METHODS

### Cell Culture and Viruses

Vero E6 cells (C1008; African green monkey kidney cells) were obtained from Public Health England and maintained in Dulbecco’s minimal essential medium (DMEM) containing 10% fetal bovine serum (FBS) and 0.05 mg/mL gentamycin at 37°C with 5% CO_2_. SARS-CoV-2 isolate SARS-CoV-2/human/Liverpool/REMRQ0001/2020, which was cultured from a nasopharyngeal swab from a patient, was passaged a further 4 times in Vero E6 cells. The fourth passage of virus was cultured in Vero E6 cells with DMEM containing 4% FBS and 0.05 mg/mL gentamycin at 37°C with 5% CO_2_ and was harvested 48 hours post inoculation. Virus stocks were stored at −80°C.

### Virus Inactivation

Heat, detergent, and UV inactivations were performed with 1.1 × 10^7^ plaque forming units (PFU) of virus. Inactivations with Trizol and Trizol LS were performed with 2.0 × 10^5^ PFU. A multiplicity of infection of 0.001 was used to infect cells destined for fixation using 100% ice-cold methanol or 4% paraformaldehyde. Cells were incubated with virus for 20 minutes at 37°C and 5% CO_2_, prior to the addition of DMEM (4% FBS, 0.05 mg/L gentamycin) and incubated for 48 hours. Heat inactivation was performed by incubating 300 µL of SARS-CoV-2 stock at 80°C for 1 hour. For inactivation with detergents, 0.5% sodium dodecyl sulfate [SDS], 0.5% Triton X-100, 0.5% Tween 20, or 0.5% NP-40 were incubated with virus for 30 minutes at room temperature. Inactivation with Trizol and Trizol LS used a 1:4 ratio of virus:Trizol reagent, which was mixed with the virus load and incubated at room temperature for 5 minutes. For fixation, after 48 hours the medium was removed and cells were removed by scraping. Cell scrapings were mixed 1:1 with the fixative for 30 minutes at room temperature. This was then centrifuged at 2500*g* for 5 minutes and the supernatant removed. Cell pellets were washed 2 times in phosphate-buffered saline (PBS) prior to resuspension up to 300 µL in PBS. UV inactivation was performed using a CL1000 UVP Crosslinker inside a microbiological safety cabinet. The CL-1000 UVP Crosslinker consisted of 5 × 254 nm shortwave tubes, which is the recommended wavelength for inactivating viruses, in particular SARS-CoV [10]. Virus stock was added (250 µL) into wells of a 24-well plate and placed without its lid on top of an ice block inside the crosslinker. Plates were placed exactly 6 cm below the UV bulbs (distance measured from the bottom of the well to the bulbs). Inactivation was performed at a range of UV energy exposures (0.01 J/cm^2^–0.8 J/cm^2^). All inactivation procedures were performed in triplicate, with the exception of NP-40 which was performed in duplicate.

### Virus Viability and Quantification

Heat-treated samples were evaluated for viable virus in a 50% tissue culture infectious dose (TCID_50_) assay with Vero E6 cells, using the entire volume of the sample. Control virus stocks containing 10^0^, 10^1^, and 10^2^ PFU incubated at room temperature for 1 hour were used to determine the limit of detection of the assay. TCID_50_ plates were passaged onto fresh cells for 4 days at least twice to ensure no replicative virus remained. Cells were monitored daily for cytopathic effect (CPE).

Virus samples treated with SDS and Triton X-100 were added to 15 mL of DMEM in a centrifugal concentrator (Amicon Ultra-15 100 kDa MWCO) and centrifuged until ≤300 µL of sample remained. Virus samples treated with Tween 20 and NP-40 were diluted in 50 mL of PBS and concentrated until ≤300 µL of sample remained. Virus samples treated with Trizol reagents were diluted in 40 mL of PBS and concentrated until ≤300 µL of sample remained. Sample was extracted and virus culture medium was added to a final volume of 300 µL. Viable virus was evaluated in a TCID_50_ assay as outlined above. Control virus stocks containing 10^0^, 10^1^, and 10^2^ PFU were diluted in PBS and followed the above protocol with centrifugal filters to determine the limit of detection of the assay. To examine the direct CPE of unfiltered detergent on the cells, additional controls with 0.05% of each detergent in the absence of virus were explored using TCID_50_. To determine if any viable virus remained in fixed cells, TCID_50_ assays were performed alongside an unfixed control. Plates were observed for CPE for 3 days and subsequently passaged onto fresh cells at least twice to ensure no virus remained. Plaque assays and TCID_50_ assays were performed on untreated virus stocks and on UV-inactivated stocks in parallel. TCID_50_ plates were passaged onto fresh cells for 3 days at least twice to ensure no replicative virus remained. TCID_50_ results were calculated using the Spearman and Karber method as previously described [11].

## RESULTS

**Figure 1.**
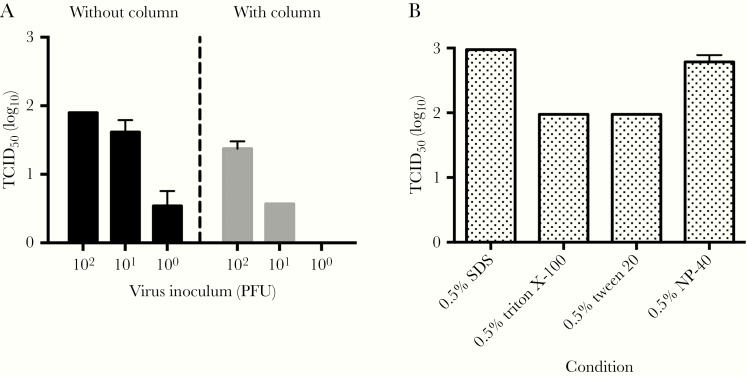
Limit of detection of SARS-CoV-2 by different methods. *A*, Assay to quantify the limits of detection. Known titers of virus were prepared with (right) or without (left) concentrating the sample in a centrifugal column. Quantification of controls was performed using TCID_50_. Lower limit of detection was 3.16 TCID_50_/mL: (n = 3; mean ± SEM). *B*, Cytotoxicity from uninfected control samples diluted in a TCID_50_ assay (n = 3; mean ± SEM). Abbreviations: PFU, plaque-forming unit; SARS-CoV-2, severe acute respiratory syndrome coronavirus 2; SDS, sodium dodecyl sulfate; SEM, standard error of the mean; TCID_50_, 50% tissue culture infectious dose.

We first determined the limits of sensitivity for our detection method by quantifying SARS-CoV-2 at 10^0^, 10^1^, or 10^2^ PFU using TCID_50_ assays. This was done by quantifying viral titers directly or by passing the sample through a centrifugal column, which is used to remove the inactivation agent before assaying on cells. Virus prepared without the centrifugal column was detected down to dilutions of 10^0^ PFU of SARS-CoV-2 (Figure 1A). The limit of detection with the centrifugal columns was determined to be 10^1^ PFU of SARS-CoV-2 (Figure 1A). Using uninfected samples containing detergents directly in the TCID_50_ assay resulted in CPE, raising the limit of detection (Figure 1B). Thus, the use of centrifugal columns to remove cytotoxic compounds allows a lower limit of detection to confirm inactivation of the virus.

**Figure 2.**
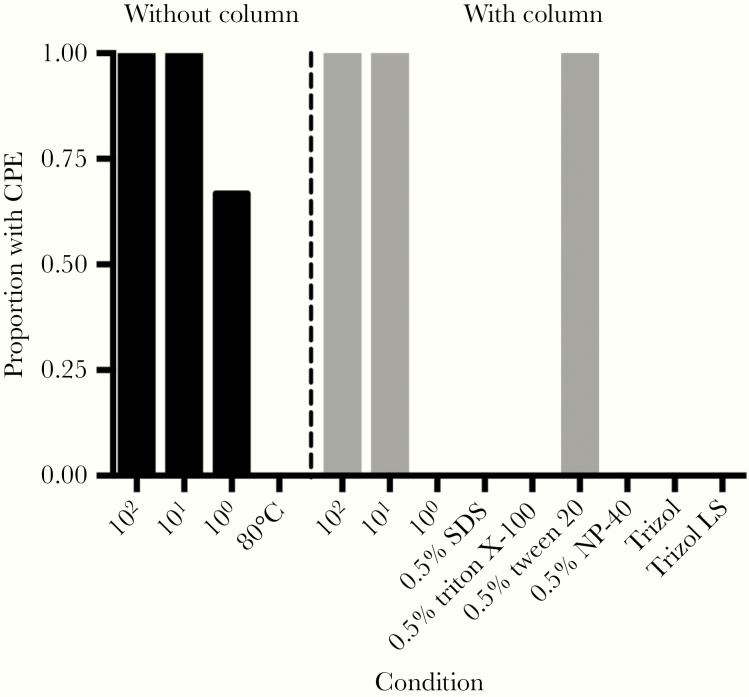
The proportion of SARS-CoV-2 inactivation assays with CPE. Samples were either diluted for assays (left) or inactivation agent removed using centrifugal columns (right). Control samples with 10^0^, 10^1^, and 10^2^ PFU of SARS-CoV-2 were used as positive controls and to determine the limit of detection for each method. Abbreviations: CPE, cytopathic effect; PFU, plaque-forming unit; SARS-CoV-2, severe acute respiratory syndrome coronavirus 2; SDS, sodium dodecyl sulfate.

**Figure 3.**
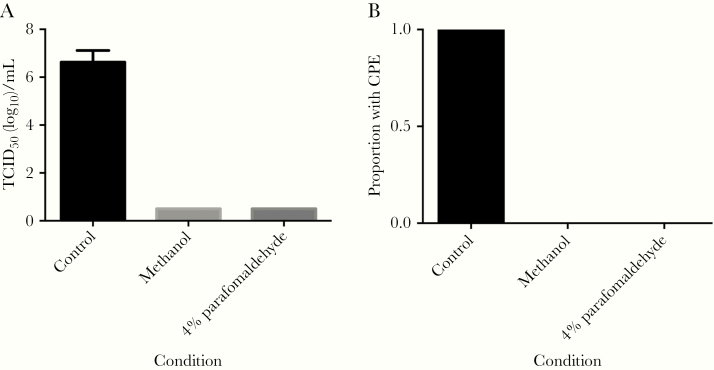
Effect of fixation on infectious virus in cells. *A*, TCID_50_ assay comparing untreated infected cells with fixed infected cells. Lower limit of detection was 0.5 TCID_50_ (log_10_)/mL (n = 3; mean ± SEM). *B*, The proportion of inactivation assays with CPE. Abbreviations: CPE, cytopathic effect; SEM, standard error of the mean; TCID_50_, 50% tissue culture infectious dose.

We then quantified virus after inactivation. SARS-CoV-2 treated at 80°C for 1 hour was successfully inactivated. We passaged samples a second time to confirmed complete viral inactivation. Using detergents and Trizol reagents, complete inactivation was seen in all replicates of SDS, Triton X-100, NP-40, Trizol, and Trizol LS, which we again confirmed by passaging the supernatant to a fresh monolayer of cells to check for residual virus (Figure 2). However, we found virus samples treated with Tween 20 all remained infectious. At the 10^0^ PFU dilution, CPE was observed in 2 of 3 replicates (Figure 2); however, virus was not detected in this dilution when using the centrifugal columns for clean-up. CPE was seen in all other control wells for either treatment. In order to determine if fixation of infected cells using methanol or paraformaldehyde resulted in virus inactivation, we performed TCID_50_ assays using unfixed infected cells and fixed cells. Complete inactivation was observed for both methanol and 4% paraformaldehyde fixed cells (Figure 3).

**Figure 4.**
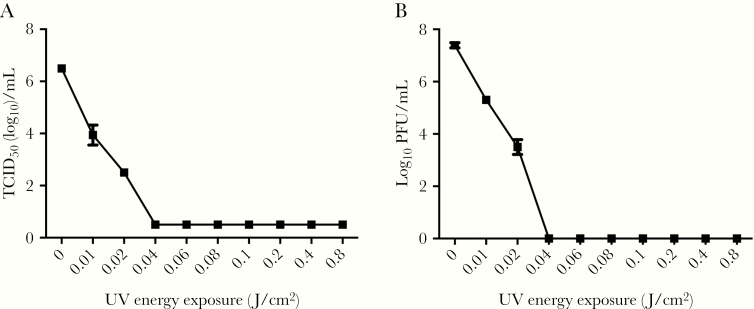
Quantification of SARS-CoV-2 following exposure to different energies of UV light. *A*, The concentration of viable SARS-CoV-2 following exposure to UV light measured by TCID_50_ assay. Lower limit of detection was 0.5 TCID_50_(log_10_)/mL. *B*, The concentration of viable SARS-CoV-2 following exposure to UV measured by plaque assay. Both assays confirmed the complete inactivation of the sample after exposure to 0.04 J/cm2 or higher UV energy. Results are expressed as mean ± SEM (n = 3). Abbreviations: PFU, plaque-forming unit; SEM, standard error of the mean; TCID_50_, 50% tissue culture infectious dose.

In order to determine if UV exposure at 254 nm would inactivate SARS-CoV-2, virus stocks were placed in wells of a 24-well plate placed on ice and exposed to varying amounts of UV energy (J/cm^2^). Exposure of SARS-CoV-2 to UV light at 0.01 J/cm^2^ resulted in partial inactivation and this increased with greater UV energy exposure, resulting in complete inactivation at UV energy exposures of more than 0.04 J/cm^2^ (Figure 4). A similar inactivation curve was seen by both TCID_50_ and plaque assay. No CPE was evident in further passages in samples where inactivation was observed.

## DISCUSSION

Virus inactivation can be achieved by several methods. However, specific methods must be chosen to comply with requirements for subsequent downstream experiments. We used a range of techniques that are often used for preserving antigens or immunological proteins to evaluate their ability to inactivate SARS-CoV-2, including a range of common detergents and determining the threshold of inactivation by UV exposure. The assay to assess infectious particles was also shown to be sensitive, in some cases down to a single infectious virus particle (TCID_50_ = 0.69 PFU/mL), and down to 10 infectious particles/mL where a centrifugal column is used to concentrate the sample.

Physical inactivation can be performed using heat or exposure to UV. Heat inactivates the virus by denaturing the structure of the proteins, affecting the attachment and replication of the virus in the host cell [12]. In this study, SARS-CoV-2 was successfully inactivated with a temperature of 80°C. Lower temperatures used to inactivate SARS-CoV showed that 56°C is only effective in the absence of fetal calf serum and temperatures up to 75°C are needed for successful inactivation of infected clinical samples [9, 10]. However, heat inactivation is not recommended in a clinical setting for immunological assays because it can interfere with the analysis of antibodies against SARS-CoV-2 [13] and diagnosis of patient samples using RT-PCR, which could potentially lead to a false-negative diagnosis [14, 15].

UV light causes genetic damage by inducing pyrimidine dimers or by producing reactive oxygen species [16]. While other researchers have investigated UV inactivation of viruses by looking at length of exposure, here we have inactivated virus based on the energy exposure. As UV lamps age, their irradiance output begins to decline. The crosslinker in this study has an inbuilt sensor allowing the unit to determine the exact amount of UV energy delivered. Therefore, to maintain consistency in experiments over time, it is recommended to inactivate virus based on the UV energy exposure rather than time of exposure. UVC exposure at 3 cm for 15 minutes has been shown to inactivate SARS-CoV, whereas UVA light was not effective [10, 17]. Here, we have demonstrated a method by which SARS-CoV-2 can be rendered noninfectious through application of UV energy >0.04 J/cm^2^. Nevertheless, we recommend that for any changes in cell substrate, virus, or for new operators, the validation process is repeated to ensure that no infectious virus remains after UV inactivation.

Chemical inactivation can be performed using detergents and Trizol reagents, and we successfully demonstrated this with 5 different compounds: 0.5% SDS, 0.5% Triton X-100, 0.5% NP-40, Trizol, and Trizol LS. Conversely, Tween 20 did not inactivate SARS-CoV-2 under the same conditions. Detergents disrupt the lipid coat of enveloped viruses and are often present in lysis buffers of commercial nucleic acid extraction kits. These detergents typically do not affect proteins so they can be used in downstream procedures preserving their native structure. Our findings are consistent with previous studies showing that 0.1% SDS with 0.1% NP-40 [10] and 0.3% tri(n-butyl)phosphate (TNBP) with 1.0% Triton X-100 [8] could inactivate SARS-CoV. Recent studies on SARS-CoV-2 showed that several lysis buffers from extraction kits like ATL (1%–10% SDS) and VXL (30%–50% guanidine hydrochloride and 1%–10% Triton X-100) from Qiagen [14] and others containing guanidine hydrochloride [18] and guanidinium [19] inactivated the virus. Several RNA extraction kits contain a lysis buffer effective at inactivating SARS-CoV-2 [20]. This is convenient for downstream experiments like qRT-PCR used for diagnosis. However, not all the laboratories may have access to these kits. The use of centrifugal columns to remove cytotoxic compounds has been successfully employed in this study, correlating to previous results [5, 21]; however, this raises the threshold of detection by approximately 10 fold. An alternative method is to dilute detergent to levels that are no longer cytotoxic. However, in our hands, this decreased our ability to detect infectious virus by 100–1000 fold. Therefore, our method using centrifugal columns was more sensitive.

Fixation using 100% methanol or 4% paraformaldehyde is often used for downstream immunohistochemical techniques or methods that require stabilization of the cellular structure. While methanol acts to precipitate large proteins and will permeabilize cells allowing the assessment of intracellular structures [22], formaldehyde reacts with amines found on proteins and nucleic acids and forms stable methylene bridge crosslinks, further preserving cell structure but potentially blocking epitopes [23]. Here we have shown that both techniques can also inactivate SARS-CoV-2, rendering fixed samples safe to process in CL2 conditions.

With the increasing interest in COVID-19, many researchers are now applying their knowledge and expertise to different topics to address this global problem. However, not all researchers have access to containment facilities and essential equipment is not often available at biosafety levels required to work safely with SARS-CoV-2. The inactivation methods described here will contribute to help diverse research groups perform their downstream work on SARS-CoV-2.

## Notes

***Acknowledgments.*** We thank Tom Curry and David Matthews for their gift of the SARS-CoV-2/human/Liverpool/REMRQ0001/2020 isolate. This isolate is freely available upon request.

***Disclaimer***. The views expressed are those of the author(s) and not necessarily those of the NHS, the National Institute for Health Research , the Department of Health, or Public Health England.

***Financial support***. This work was supported by the Liverpool School of Tropical Medicine Director’s Catalyst Fund Award (to E. I. P. and A. C.-S.); Biotechnology and Biological Sciences Research Council (grant numbers BB/T001240/1 to G. L. H. and BB/S0011980/1 to A. C.-S. and A. A.-S.); Royal Society Wolfson Fellowship (grant number RSWF\R1\180013 to G. L. H.), National Institutes of Health (grant numbers R21AI138074 and R21AI129507 to G. L. H.); UK Research and Innovation (grant number 20197 to G. L. H.); National Institute for Health Research (grant number NIHR2000907 to G. L. H.); Medical Research Council Developmental Pathway Funding Scheme (grant number MR/R015406/1 to T. P. and M. J. G.); Wellcome (grant number 205228/Z/16/Z Clinical Career Development Fellowship to L. T.); and the DogsTrust (to S. L. S.). G. L. H., T. P., T. S., and L. T. are affiliated to the National Institute for Health Research Health Protection Research Unit (NIHR HPRU) in Emerging and Zoonotic Infections at University of Liverpool in partnership with Public Health England (PHE), in collaboration with Liverpool School of Tropical Medicine and the University of Oxford. G.L.H. is based at LSTM and T. P., T. S., and L. T. at the University of Liverpool. The views expressed are those of the author(s) and not necessarily those of the NHS, the NIHR, the Department of Health or Public Health England.

***Potential conflicts of interest.*** All authors: No reported conflicts of interest. All authors have submitted the ICMJE Form for Disclosure of Potential Conflicts of Interest. Conflicts that the editors consider relevant to the content of the manuscript have been disclosed.

## References

[1] Wu F, Zhao S, Yu B, et al. A new coronavirus associated with human respiratory disease in China. Nature 2020; 579:265–9. 32015508 10.1038/s41586-020-2008-3 PMC7094943

[2] Zhou P, Yang XL, Wang XG, et al. A pneumonia outbreak associated with a new coronavirus of probable bat origin. Nature 2020; 579:270–3. 32015507 10.1038/s41586-020-2012-7 PMC7095418

[3] World Health Organization. WHO Coronavirus disease (COVID-19) dashboard https://covid19.who.int. Accessed 13 August 2020.

[4] Lu R, Zhao X, Li J, et al. Genomic characterisation and epidemiology of 2019 novel coronavirus: implications for virus origins and receptor binding. Lancet 2020; 395:565–74. 32007145 10.1016/S0140-6736(20)30251-8 PMC7159086

[5] Patterson EI, Warmbrod KL, Bouyer DH, Forrester NL Evaluation of the inactivation of Venezuelan equine encephalitis virus by several common methods. J Virol Methods 2018; 254:31–4. 29407211 10.1016/j.jviromet.2018.01.009 PMC5826796

[6] Song H, Li J, Shi S, Yan L, Zhuang H, Li K Thermal stability and inactivation of hepatitis C virus grown in cell culture. Virol J 2010; 7:40. 20167059 10.1186/1743-422X-7-40 PMC2834657

[7] Wang J, Mauser A, Chao SF, et al. Virus inactivation and protein recovery in a novel ultraviolet-C reactor. Vox Sang 2004; 86:230–8. 15144527 10.1111/j.0042-9007.2004.00485.x

[8] Rabenau HF, Biesert L, Schmidt T, Bauer G, Cinatl J, Doerr HW SARS-coronavirus (SARS-CoV) and the safety of a solvent/detergent (S/D) treated immunoglobulin preparation. Biologicals 2005; 33:95–9. 15939287 10.1016/j.biologicals.2005.01.003 PMC7128630

[9] Rabenau HF, Cinatl J, Morgenstern B, Bauer G, Preiser W, Doerr HW Stability and inactivation of SARS coronavirus. Med Microbiol Immunol 2005; 194:1–6. 15118911 10.1007/s00430-004-0219-0 PMC7086689

[10] Darnell ME, Subbarao K, Feinstone SM, Taylor DR Inactivation of the coronavirus that induces severe acute respiratory syndrome, SARS-CoV. J Virol Methods 2004; 121:85–91. 15350737 10.1016/j.jviromet.2004.06.006 PMC7112912

[11] Hierholzer J, Killington R Virus isolation and quantitation. In: Mahy B, Kangro H, eds. Virology methods manual. San Diego, CA: Academic Press, 1996:24–32.

[12] Perdiz D, Gróf P, Mezzina M, Nikaido O Distribution and repair of bipyrimidine photoproducts in solar UV-irradiated mammalian cells. Possible role of Dewar photoproducts in solar mutagenesis. J Biol Chem 2000; 275: 26732–42. 10827179 10.1074/jbc.M001450200

[13] Hu X, An T, Situ B, et al. Heat inactivation of serum interferes with the immunoanalysis of antibodies to SARS-CoV-2 [published online ahead of print 28 June 2020]. J Clin Lab Anal doi: 10.1002/jcla.23411. PMC7361150 32594577

[14] Pastorino B, Touret F, Gilles M, de Lamballerie X, Charrel RN Evaluation of heating and chemical protocols for inactivating SARS-CoV-2. Viruses 2020; 12:735. 10.3390/v12060624 PMC7354533 32521706

[15] Pan Y, Long L, Zhang D, et al. Potential false-negative nucleic acid testing results for severe acute respiratory syndrome coronavirus 2 from thermal inactivation of samples with low viral loads. Clin Chem 2020; 66:794–801. 32246822 10.1093/clinchem/hvaa091 PMC7184485

[16] Ravanat JL, Douki T, Cadet J Direct and indirect effects of UV radiation on DNA and its components. J Photochem Photobiol B 2001; 63:88–102. 11684456 10.1016/s1011-1344(01)00206-8

[17] Kariwa H, Fujii N, Takashima I Inactivation of SARS coronavirus by means of povidone-iodine, physical conditions and chemical reagents. Dermatology 2006; 212(suppl 1):119–23. 16490989 10.1159/000089211 PMC7179540

[18] Nelson AC, Auch B, Schomaker M, et al. Analytical validation of a COVID-19 qRT-PCR detection assay using a 384-well format and three extraction methods. bioRxiv 022186 [Preprint]. 2 April 2020 [cited 13 August 2020]. Available from: 10.1101/2020.04.02.022186.

[19] Grant PR, Turner MA, Shin GY, Nastouli E, Levett LJ Extraction-free COVID-19 (SARS-CoV-2) diagnosis by RT-PCR to increase capacity for national testing programmes during a pandemic. bioRxiv 028316 [Preprint]. 6 April 2016 [cited 13 August 2020]. Available from: 10.1101/2020.04.06.028316.

[20] Centers for Disease Control and Prevention. CDC 2019-novel coronavirus (2019-nCoV) real-time RT-PCR diagnostic panel, 2020 https://www.fda.gov/media/134922/download. Accessed 13 August 2020.

[21] Bergren NA, Patterson EI, Blair H, Ellis RP, Kading RC Methods for successful inactivation of Rift Valley fever virus in infected mosquitoes. J Virol Methods 2020; 276:113794. 31794780 10.1016/j.jviromet.2019.113794

[22] Hobro AJ, Smith NI An evaluation of fixation methods: spatial and compositional cellular changes observed by Raman imaging. Vib Spectrosc 2017; 91:31–45.

[23] Shi SR, Cote RJ, Taylor CR Antigen retrieval immunohistochemistry: past, present, and future. J Histochem Cytochem 1997; 45:327–43. 9071315 10.1177/002215549704500301

